# Development of Language Models for Continuous Uzbek Speech Recognition System

**DOI:** 10.3390/s23031145

**Published:** 2023-01-19

**Authors:** Abdinabi Mukhamadiyev, Mukhriddin Mukhiddinov, Ilyos Khujayarov, Mannon Ochilov, Jinsoo Cho

**Affiliations:** 1 Department of Computer Engineering, Gachon University, Sujeong-gu, Seongnam-si 13120, Republic of Korea; 2 Department of Information Technologies, Samarkand Branch of Tashkent University of Information Technologies Named after Muhammad al-Khwarizmi, Tashkent 140100, Uzbekistan; 3 Department of Artificial Intelligence, Tashkent University of Information Technologies Named after Muhammad al-Khwarizmi, Tashkent 100200, Uzbekistan

**Keywords:** language model, Uzbek speech, recurrent neural networks, automatic speech recognition, neural networks, character-based language models, word-based language models

## Abstract

Automatic speech recognition systems with a large vocabulary and other natural language processing applications cannot operate without a language model. Most studies on pre-trained language models have focused on more popular languages such as English, Chinese, and various European languages, but there is no publicly available Uzbek speech dataset. Therefore, language models of low-resource languages need to be studied and created. The objective of this study is to address this limitation by developing a low-resource language model for the Uzbek language and understanding linguistic occurrences. We proposed the Uzbek language model named UzLM by examining the performance of statistical and neural-network-based language models that account for the unique features of the Uzbek language. Our Uzbek-specific linguistic representation allows us to construct more robust UzLM, utilizing 80 million words from various sources while using the same or fewer training words, as applied in previous studies. Roughly sixty-eight thousand different words and 15 million sentences were collected for the creation of this corpus. The experimental results of our tests on the continuous recognition of Uzbek speech show that, compared with manual encoding, the use of neural-network-based language models reduced the character error rate to 5.26%.

## 1. Introduction

To provide probabilistic predictions for the next word in a sequence of words in natural speech, a language model (LM) is the primary function or learning approach. Continuous speech recognition requires an LM to be built into an efficient system. An LM is essential for many applications in natural language processing (NLP), including but not limited to handwriting recognition [1,2,3,4], machine translation [5], speech recognition [6,7], integral model [8], phonemes [9], and vowels [10].

In the absence of an LM, the capabilities of speech recognition approaches that rely on methods such as grammar are severely constrained. The N-gram statistical model is one of the most widely used language models. Although such LMs achieve robust results when applied to languages with a fixed word order (such as English), they need to be enhanced when used with the Uzbek language, which has a more flexible word order [11]. To tackle the problem of the automatic recognition of continuous Uzbek speech, we present the design and implementation of a recurrent neural network (RNN) based LM.

Artificial neural networks (ANNs), such as feedforward and recurrent models, are widely employed in an LM. Furthermore, LMs are used in the development of smart communication systems that exploit enormous libraries of recorded human data to better predict and understand the world. There are various uses of automated speech recognition (ASR), in which only the lowest portion of speech and manually reviewed text transcriptions are given open access for the training and modification of the LMs applied. Natural language communication facilitates the high-level cognition required for successful idea sharing between humans and artificial intelligence. Language adaptability makes it possible to describe a wide range of intelligence-related operations, including the analysis and generation of natural languages. Distributed representations and extended contexts are two benefits of language models. RNNs are promising models for neural language modeling owing to their unique dynamics, which permits data recycling within a network. The limitations of a regular RNN in affecting the extended contexts are addressed using the long short-term memory (LSTM) approach. Despite numerous RNN alternatives, the potential of including multiple memory cells in LSTM nodes has yet to be explored.

Owing to recent advancements in machine learning, neural language models can now conduct more sophisticated NLP tasks, including sentiment analysis and post-tagging. Many high-level occupations are currently available, including call centers, question-answering, discussion, and fact-checking systems. As a result of using a pretrained language model, significant progress in NLP has been achieved [12,13]. Most state-of-the-art results to NLP problems have been accomplished using massive unsupervised learning with large plain texts trained with contextual language representations. However, most research in the field of computational linguistics is limited to well-known languages. When creating a language model for a language with a smaller body of study, such as Uzbek, it is crucial to pay close attention to the linguistics of the language. However, Uzbek is unlike other languages because of its unusual structure. As a result, modeling of the Uzbek language is a challenge.

Predicting the next word in a sequence is a common training goal for models of all languages [14]. However, this approach does not apply to the Uzbek language, which requires a strict subject–object–verb structure. Sentences in languages with this structure are typically organized such that information appear at the end. The systematic lack of order in Uzbek makes language modeling even more of a challenge, making it difficult, if not impossible, to predict the next word. To explain the distinctive architecture of the Uzbek language, it is therefore necessary to develop a novel training technique.

Although studies on multilingual language models, such as Multilingual BERT, have been conducted, a more profound and familiar study of Uzbek language modeling remains a possibility. Current language models that can be modified into different languages have extensive linguistic resources and an exceptional performance. Multilingual BERT is worse than the English version, and most studies on pretrained language models cover only English [15]. Recent studies in the field of language modeling include ELECTRA [16], Xlnet [17], BERT [12], and BART [13], all of which were trained in English. It is therefore necessary to develop a modified language model for Uzbek.

Data resources regarding the Uzbek language are limited. Written materials on the Internet can be used as a sufficient training corpus for English language modeling. Although Wikipedia articles and other knowledge-rich corpora are commonly used for the pre-training of language models, the number of articles on Wikipedia varies widely between languages [12]. This means that for lesser-studied languages, it is either impossible or extremely difficult to compile a substantial corpus from the content found on the web. Because many people use languages other than English as their mother tongue, it is vital to develop a language model for low-resource languages, even if little information is available to them. In light of this, we should prioritize the realistic training of a Uzbek language model using smaller model sizes and fewer training data, rather than leveraging enormous numbers of data and high processing capabilities.

To construct a statistical LM, we need access to a large text corpus (database), which is a collection of texts in one or more languages with associated parameters [18]. In this study, a text corpus from several digital media forms was employed to train neural networks and establish a baseline for trigram LMs. In the speech decoding step, a trigram LM was used to create a list of N-best recognition hypotheses (N-best list), that is, a text list consisting of N hypotheses with the most considerable probabilities gained through linguistic or acoustic models. We then rescored these hypotheses, rearranging them based on probability estimates, and computed new estimates for this set using an RNN-based LM. We applied a linear interpolation between the LM generated by the neural network and that generated by the trigram. The interpolation of language models is the process of integrating word probabilities from many models using a linear technique, while providing each model with various weights. We employed a separate test corpus of continuous Uzbek speech to evaluate the effectiveness of a neural-network and LM-based automatic speech recognition system [19]. The speech corpus is an organized collection of spoken audio data.

This study proposes the use of several integrated neural network models for the building of statistical and neural-network-based language models on ASR networks for the Uzbek language and its dialects. In this case, an RNN encoder–decoder, DNN-CTC, E2E-Transformer, and E2E-Conformer were used.

The following are the most important findings from the present research:

- A 105 h speech corpus was created for the development of large-scale continuous speech recognition systems in the Uzbek language.
- Language models based on statistics and neural networks have been created for continuous speech recognition in the Uzbek language. The perplexity index in the developed language models was 7.2 in the 3-gram language model and 2.56 in the LSTM language model.
- Without an LM, the E2E-Transformer model achieved a WER of 34.1% and a CER of 12.1% on the training set, and a WER of 30.9% and a CER of 8.9% on the test set. By combining the developed language model with a speech recognition system, a WER of 23.9% and a CER of 11.0% occur in the training sample, and a WER of 22.5% and a CER of 8.5% are found in the test sample.

The remainder of this paper is organized as follows. Section 2 examines some of the most widely used methods. Section 3 describes the proposed LM types for the Uzbek language. The implementation and evaluation of the proposed system using alternative state-of-the-art methods are detailed in Section 4, respectively. Finally, Section 5 presents the key results.

## 2. Related Work

The use of ANNs in language modeling was pioneered by Schwenk et al. [20], who evaluated an ANN-based N-gram model against an improved Kneser–Ney smoothing method utilizing an LM which is learned data from a corpus including more than 550 million words. The most common words were chosen for inclusion in the ANN-based LM rather than a complete vocabulary. The authors suggested a technique for training a neural network using a vast training corpus. A different portion of the text was randomly chosen during each training iteration. An N-gram LM was employed for speech recognition, whereas an LM based on neural network was used to rescore the word lattice. A 0.5% drop in misidentified words occurred. To speed up the training process, Mikolov et al. [21] introduced an RNN model to create an LM by merging rare words into a new category (words that occur below a certain threshold number). In the speech recognition experiments, a 5-gram LM based on Kneser–Ney smoothing served as the baseline, whereas an LM based on RNN was utilized to rescore the top-100 hypotheses. Owing to the use of an RNN, the word error rate (WER) was reduced by 18% compared to the 5-gram LM, and the complexity of the LM was reduced by 5%.

Huang et al. [22] proposed an LM based on the use of an RNN for the initial decoding stage of a Bing voice search. It was proposed that the LM based on RNN model only used if the N-gram LM estimate for the predicted word is sufficiently high. The authors employed a key-value hash table cache to speed up the processing. This LM can reduce the WER from 25.3% to 23.2%. Recognition lattice reweighting was also applied using an RNN-based LM. The best results were achieved when an LM based on RNN was connected with a baseline 4-gram model to generate the lattice. The lattice was subsequently rescored using similar model, with an interpolation coefficient of 0.3, and obtained 22.7% of the WER value. Sundermeyer et al. [23] examined the differences between LMs fabricated with feedforward ANNs and those fabricated using RNNs. The authors tested three different neural network LM arrangements: (1) the use of the LIMSI program to build a feedforward ANN with a boundary defined based on the most frequently occurring words in the output layer, (2) clustering technique with a feedforward ANN (where the complete word pair is utilized), and (3) clustering with RNN. The LM was trained using 27 million words. Based on the frequency distribution of the words, 200 classes were established for ANN clustering. As the results of the validation data indicate, the size of hidden layer was changed from 300 to 500 units. An N-gram model was applied to extrapolate the LM from the ANN-based system. Compared with the baseline system, the most significant absolute reduction in the WER was 1.5% for the training set and 1.4% for the test set. In the experiments, LMs built using feedforward ANNs performed worse than those built using RNNs. The RNN performed 0.4% better on the test data than the feedforward ANN. Morioka et al. [24] proposed an LM that takes advantage of a variable-length context. Speech recognition experiments using an extensive dictionary revealed that this model is superior in terms of a reduced perplexity and WER values.

To better utilize alternate uncertain ASR hypotheses obtained from constrained amounts of in-domain speech, Sheikh et al. [25] recently investigated the training and adaptation of LSTM RNN LMs using ASR confusion networks. The authors presented three approaches: (1) Kullback–Leibler (KL) divergence loss, (2) a hidden Markov model (HMM) formulation, and (3) path sampling from the confusion networks. Compared with training on ASR 1-best transcripts, the sampling-based method, and in some circumstances the KL divergence method, led to substantial declines in perplexity. In 2022 [26], the authors applied the findings of [25] to a Transformer LM. On multiple large- and medium-sized ASR benchmarks, Transformer LMs outperformed LSTM LMs [27]. It was found experimentally that the self-attention modules of Transformer LMs at various levels can pick up not only global information and instance-specific patterns but also the local N-gram-like context [28]. It would be interesting to test the self-attention mechanism of the Transformer to determine whether it can improve the performance of LSTM LMs under constrained data settings by taking advantage of alternative hypotheses provided by ASR confusion networks. To improve the performance of ASR rescoring, machine translation, and spoken language understanding tasks, previous studies have extended the use of the Transformer to include confusion networks [29,30] and ASR lattices [31,32]. A Transformer encoder was utilized in these studies to incorporate the lattice or confusion network into vector representations that can be used for rescoring, classification, or translation. By contrast, the training of Transformer LMs on ASR-decoded graphs is also tricky because the input and output targets at each stage of a word sequence are not a single class or word but rather a series of indefinite word hypotheses.

Recently, UzBERT is a BERT-based, pre-trained Uzbek language model recently introduced by Mansurov et al. [33]. The authors created a high-quality news corpus of more than 142 M words, pre-trained a model, and compared its accuracy in masked language to that of multilingual BERT (mBERT). They adopted training objectives (MLM and NSP), hyperparameters like dropout probability 0.1 and a GeLU activation, and the vocabulary size of 30 K tokens are all part of the original BERT’s network architecture of 12 layers, 768 hidden dimensions, 12 attention heads, and 110 M parameters. In total, there are 142 M words in those articles; 140 M are used for training, and the remaining 2 M are used for validation. Although hybrid CTC/attention ASR systems have gained popularity and improved significantly even in low-resource environments, they are rarely used for Central Asian languages like Turkish and Uzbek. Ren et al. [34] proposed a CNN-based feature extractor called Multi-Scale Parallel Convolution (MSPC) that uses different convolution kernel sizes to extract features of different sizes and combined it with bidirectional long short-term memory (Bi-LSTM) to form an encoder structure to boost the end-to-end model’s recognition rate and system robustness. The authors initialized the RNN language model with a fine-tuned pre-trained BERT and incorporated it into the decoding process. To better understand the End2End approach and how it can be used for the Uzbek language, Mamatov et al. [35] developed an Uzbek-language speech recognition system based on evaluating available speech recognition methods and selecting a preferred method. In addition to analyzing transformers, the authors trained different models with the speeches’ 432 h of audiobooks and 72 h of audio recordings of aphorisms, proverbs, and 174 speakers to create a database.

Despite the increasing interest in employing ANNs for language modeling, more research needs to be conducted regarding the Uzbek language. Herein, we describe how to build a language model of Uzbek that improves the recognition of Uzbek speech in real time.

## 3. Materials and Methods of Language Model

We expect that Expanding and diversifying the language model’s corpus will lead to more generalized ASR. USC (Uzbek Speech Corpus) (https://github.com/Smart-Projects-Artificial-Intelligence/Uzbek-ASR; https://usc.spai.uz/uz/, accessed on 1 March 2021) helps us construct the character-based language model.

The Uzbek speech corpus (USC) has been developed in collaboration between ISSAI and the Image and Speech Processing Laboratory in the Department of Computer Systems of the Tashkent University of Information Technologies. The USC comprises 958 different speakers with a total of 105 h of transcribed audio recordings (https://www.researchgate.net/publication/353634969_USC_An_Open-Source_Uzbek_Speech_Corpus_and_Initial_Speech_Recognition_Experiments, accessed on 21 September 2021). To ensure high quality, the USC has been manually checked by native speakers. The USC is primarily designed for automatic speech recognition (ASR), however, it can also be used to aid other speech-related tasks, such as speech synthesis and speech translation. To the best of our knowledge, the USC is the first open-source Uzbek speech corpus available for both academic and commercial use under the Creative Commons Attribution 4.0 International License. We expect that the USC will be a valuable resource for the general speech research community and become the baseline dataset for Uzbek ASR research. Therefore, we invited other researchers to use our dataset and helped to further explore it with us (https://www.kaggle.com/datasets/bexruznutfilloyev/voice-recognitionuzbek, accessed on 14 June 2022). Continuing collaborations, the Common Voice speech (https://huggingface.co/lucio/xls-r-uzbek-cv8, accessed on 14 June 2022) corpus was launched to train an Uzbek-automated speech recognition system using natural language processing.

Our research requires an Uzbek text corpus to create a language model. CC100-uzbek (https://metatext.io/datasets/cc100-uzbek, accessed on 18 August 2021) dataset corresponds to our study. In this case, the texts were collected using a crawler, but the preliminary processing work, redundant characters, numbers, etc., were not removed. Therefore, we did not directly use such a dataset to generate LM because it was not preprocessed.

In addition, we manually filtered the collected texts to eliminate defects peculiar to web crawlers and exclude non-Uzbek sentences and inappropriate content (e.g., user privacy and violence). We kept sentences containing borrowed words from other languages such as English and Russian.

*Creation of a text corpus.* Creating a corpus of large text is one of the most complex and time-consuming tasks. Traditional methods for extracting information from various Internet sources are time-consuming. In addition, because the collected data are presented in different ways, it is necessary to conduct data normalization procedures to bring them into a uniform format, which in turn leads to the loss of collected data. In addition, maintaining data integrity is occasionally the most important task.

**Figure 1:**
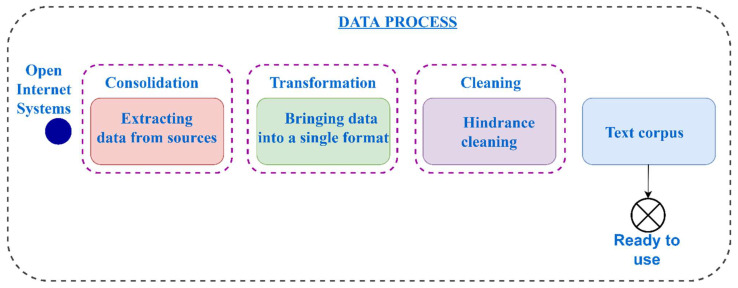
Generalized scheme of data processing.

It is generally accepted that data pre-processing can be divided into three stages: consolidation, transformation, and cleaning (Figure 1). The most complex stage is consolidation, which includes data collection. The main concept of consolidation is the data source, i.e., an object containing information necessary to solve a specific practical problem.

Data *consolidation* refers to a collection of processes that take several datasets and transform them into a uniform format suitable for usage in an analytical database or data warehouse.

Data consolidation is the first step in solving an optional project or analytical task. It is based on the process of organizing data collection and storage in the most convenient form for solving specific analytical tasks or solving a problem on a specific analytical platform.

Data consolidation should have the following optimization criteria:

➢ Providing high-speed access to information;
➢ Storage compactness;
➢ Automatic support of data structure integrity;
➢ Data consistency control.

**Figure 2:**
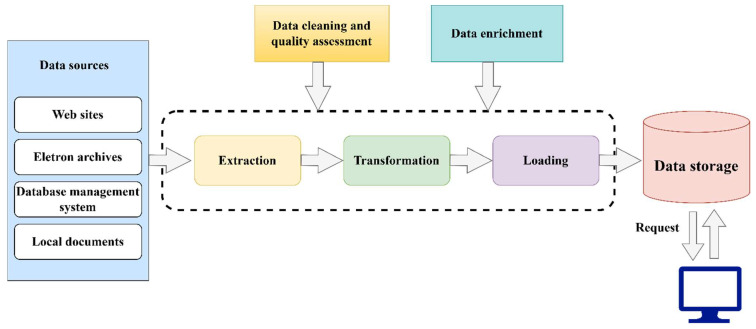
Data consolidation process.

It follows that the generalized scheme of the consolidation process can be expressed in the form of the following block diagram (Figure 2).

There are many systems used for extracting data from Internet sources [36], including TSIMMIS, WebOQL, FlORID, XWRAP, RoadRunner, Lixto, RAPIER, SRV, WHISK, and BeautifulSoup. Almost all of these systems are highly specialized for specific tasks: some are focused on working with relatively grammatically consistent texts, whereas others are associated with a specific data structure of the same type; therefore, the use of such tools is unsuitable for solving the problem. In addition to these systems, search robots (web crawlers) are used for data collection. A search robot is a special program whose main task is to collect data based on crawled websites.

A *transformation* consists of a collection of methods and algorithms designed to optimize the presentation and formats of data based on the tasks being solved and the analytics objectives. The goal of data transformation is not to alter the data’s actual content; instead, it is to display the data in a unified manner so that it may be utilized efficiently, such as, by translating numbers to text or dealing with abbreviations.

*Cleaning* is the process of identifying and eliminating various factors that interfere with the correct analysis of data, including anomalous and random values, symbols belonging to a different alphabet, and unfamiliar or repetitive texts, various paradoxes, and noises [37].

**Figure 3:**
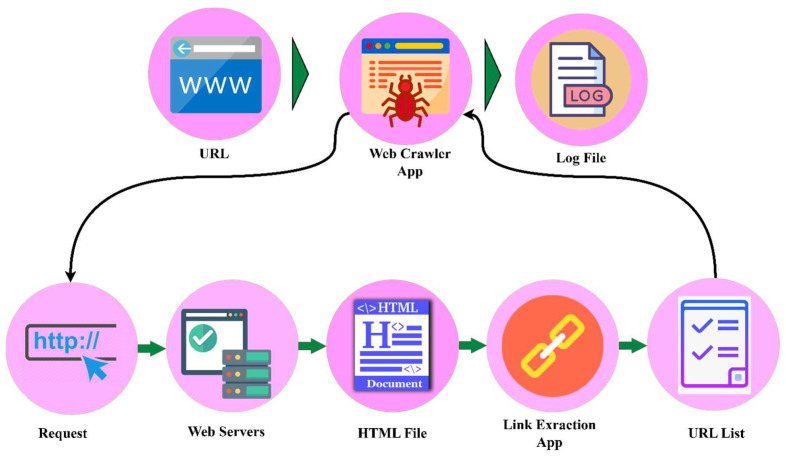
How web crawlers operate.

As a part of this study, we created a search robot (web crawler) (Figure 3) that automatically collects texts from the Uz domain at the consolidation stage to form a database of texts in the Uzbek language. In addition, when extracting data from webpages, the BeautifulSoup Python package was used, and texts consisting of more than one million sentences were collected.

In the text collection, we collected and processed a corpus of texts in the Uzbek language, which was compiled from news feeds from three recent sites: www.kun.uz, www.daryo.uz, and www.ziyouz.com, which contain the latest urgent news and news from Uzbekistan and the world. These sites contain texts that reflect the current state of Uzbek. When updating websites online, it is possible to automatically fill in the corpus of texts, which allows the addition of new words that appear in the language and retrains the language model to account for new textual information.

After bringing the collected texts into a single format (through a transformation process) and removing noise from the text (cleaning process), a text corpus comprising more hundred thousand sentences was collected. Approximately 80 million Uzbek words were used in the sentences.

**Language models**. Language modeling is an essential component in real-world applications such as machine translation and automatic speech recognition. Therefore, language modeling plays a key role in the development of applications related to NLP, computational linguistics, and speech recognition.

The purpose of language modeling is to estimate the probability distribution of various language units such as words and sentences. Language models help choose the next words in the text, taking into account the probability that the word comes before and after other words [38]. The process of speech recognition involves the problem of the transcription of a speech signal as a sequence of words. From this perspective, the role of language models in speech recognition is crucial. It is possible to find an application of the language model based on the sequence of letters [39] or the sequence of words [40]. Both are used in the development of speech-recognition systems [21,41]. In general, an approach to finding a sequence of words is widely applied.

To create a language model, several probabilistic approaches have been used. These approaches vary depending on why the language model was created. The analyzed text data and the methods applied for analysis (mathematical approaches) differ in their approaches to creating and training a language model. Predicting the next word in a search query is extremely different from identifying suitable sentences in machine translation. Speech recognition requires a special approach to creating a language model.

There are two main types of language models, i.e., language models based on statistics and language models of neural networks. 

**Language model based on statistics**. Statistical models are based on the development of probabilistic models that can predict the next word given the previous words.

A probabilistic distribution of a sequence of words is constructed using a statistical formula to describe the language model. The most prominent example of this type of model is an N-gram, which is one of the easiest tools used to model a language, and of which there are many different types, for instance, a 1-gram (unigram), 2-gram (bigram), and 3-gram (trigram).

To calculate the probability of a sentence, we use the following formula:

(1) $$\begin{array}{c} P\left(w_{1}w_{2}\ldots w_{n}\right)=\prod_{i}P\left(w_{i}|w_{1}w_{2}\ldots w_{i-1}\right). \end{array}$$

The extended form of (1) has the following form:

(2) $$\begin{array}{c} P\left(w_{1}^{n}\right)=P\left(w_{1}\right)P\left(w_{2}|w_{1}\right)P\left(w_{3}|w_{1}^{2}\right)\ldots P\left(w_{n}|w_{1}^{n-1}\right)=\prod_{k=1}^{n}P\left(w_{k}|w_{1}^{k-1}\right). \end{array}$$

According to a Markov assumption, it is sufficient if the target word is determined by *n* previous words and not by all of them. Equation (1) can then be written as follows:

(3) $$\begin{array}{c} P\left(w_{1}w_{2}\ldots w_{n}\right)\approx \prod_{i}P\left(w_{i}|w_{i-k}\ldots w_{i-1}\right), \end{array}$$

(4) $$\begin{array}{c} P\left(w_{1}w_{2}\ldots w_{i-1}\right)\approx P\left(w_{i}|w_{i-k}\ldots w_{i-1}\right). \end{array}$$

If the above expression is written for N = 2, that is, for a 2-gram model, then (4) can be written as follows:

(5) $$\begin{array}{c} P\left(w_{n}|w_{1}^{n-1}\right)\approx P\left(w_{n}|w_{n-1}\right). \end{array}$$

Here, *w_i_* are words, and *P* is a probability function found through (6). This is called a maximum likelihood estimate (MLE).

(6) $$\begin{array}{c} P\left(w_{n}|w_{n-N+1}^{n-1}\right)=\frac{C\left(w_{n-N+1\;}^{n-1}\;w_{n}\right)}{C\left(w_{n-N+1}^{n-1}\right)}. \end{array}$$

Here, *C* is a special function that counts the number of occurrences of a sentence in a text.

Example: *P(“salom”|”dunyo”) = C(“salom dunyo”)/C(“salom”)*. This example calculates the probability that the word “dunyo” will appear after the word *“salom”.* However, if the phrase *“salom dunyo”* does not occur even once in the text as an example, the value is 0. Otherwise, if the word *“salom”* is not found in the text, a calculation error will occur. To solve this problem, backoff, interpolation, and smoothing methods are used.

The backoff and interpolation algorithms are implemented because when the N-gram model is calculated, if the value of N is not satisfied (in the absence of a sequence of such a length in the text), the calculation is organized into a unigram through its reduction. In addition to the backoff algorithm, a weight value is assigned to each N-gram. Then, if we look at (6) above with N = 3, we have the following:

(7) $$\begin{array}{c} \hat{P}\left(w_{n}|w_{n-2}w_{n-1}\right)=\lambda_{1}P\left(w_{n}|w_{n-2}w_{n-1}\right)+\lambda_{2}P\left(w_{n}|w_{n-1}\right)+\lambda_{3}P\left(w_{n}\right),\; \end{array}$$

Here, the values of $\underset{i}{\sum }\lambda_{i}=1$, $\lambda_{i}$ are determined using special learning algorithms [42,43].

The backoff algorithm can be used in conjunction with interpolation.

$$\hat{P}\left(w_{i}|w_{i-2}w_{i-1}\right)=\left\{\begin{array}{c} P\left(w_{i}|w_{i-2}w_{i-1}\right),\;if\;C\left(w_{i-2}w_{i-1}w_{i}\right)>0\\ \alpha_{1}P\left(w_{i}|w_{i-1}\right),\;if\;C(w_{i-2}w_{i-1}w_{i)}=0\\ and\;C\left(w_{i-1}w_{i}\right)>0\\ \alpha_{2}P\left(w_{i}\right),\;otherwise \end{array}\right.$$

Laplace smoothing, add-k smoothing, good-turning estimate, absolute discounting, and Kneser–Ney are smoothing algorithms. Each has its own scope, advantages, and disadvantages [44]. Among them, Kneser–Ney smoothing is highly effective in the field of speech recognition. As described in [45,46,47], the use of Kneser–Ney smoothing in an N-gram language model yields excellent results. With the Kneser–Nei smoothing algorithm, the probability calculation is recursively applied using the following expression:

(8) $$\begin{array}{c} P_{KN}\left(w_{i}|w_{i-n+1}^{i-1}\right)=\frac{\max\left(c_{KN}\left(w_{i-n+1}^{i}\right)-d,0\right)}{\sum_{v}c_{KN}\left(w_{i-n+1}^{i}v\right)}+\lambda \left(w_{i-n+1}^{i-1}\right)P_{KN}\left(w_{i}|w_{i-n+2}^{i-1}\right). \end{array}$$

Here, *d* is the value of absolute discounting, which is typically chosen to be between 0 and 1.

Linear models such as N-grams do not perform well when the dataset is large and consists of rarely used or unique words. This is due to the increase in the number of words and, as a consequence, the number of possible word sequences. Consequently, predicting the next word is difficult. As another disadvantage of this model, it must use Markov assumptions in the N-gram models. In long contextual sentences, the overall probability score is considered to be assumptive. With natural language, in long contexts, each word in a sentence affects the conditional probability [48]. These give rise to the concept of using deep learning and neural networks in the building of language models.

Several other types of statistical language models allow the modeling of long contexts or long-term relationships between words. These types of statistical language models include trigger models, a simplified type of trigger model (cache model), class-based models, distance models, topic mixture models, and particle-based models [49].

**Language model of a neural network**. Neural network language models are more efficient than N-gram language models and are of several types. Examples of these include feedforward neural networks (FFNNs) [41], RNNs [50], language models based on long-term dependence to help overcome any difficulties, a language model with LSTM, and attention-based language models, which are effectively used in speech recognition.

**Language Models Based on Feed-forward Neural Networks**. The idea of using neural networks in the development of various language models was first considered in [50], where the proposed language model performs better than N-gram language models because of their low ability to generalize from a lack of hidden layers. It was therefore impossible to take into account context-dependent features.

According to (1), when calculating the conditional probability P of the language model, *w_i_* is directly dependent on *w_1_…w_i−_*_1_. This creates a problem in that the lengths of the input vectors in the neural network have different sizes. To solve this problem, it is possible to use (4) based on a Markov assumption in the N-gram model. The FFNN language model considers the previous n − 1 words as the context for predicting the next word.

**Figure 4:**
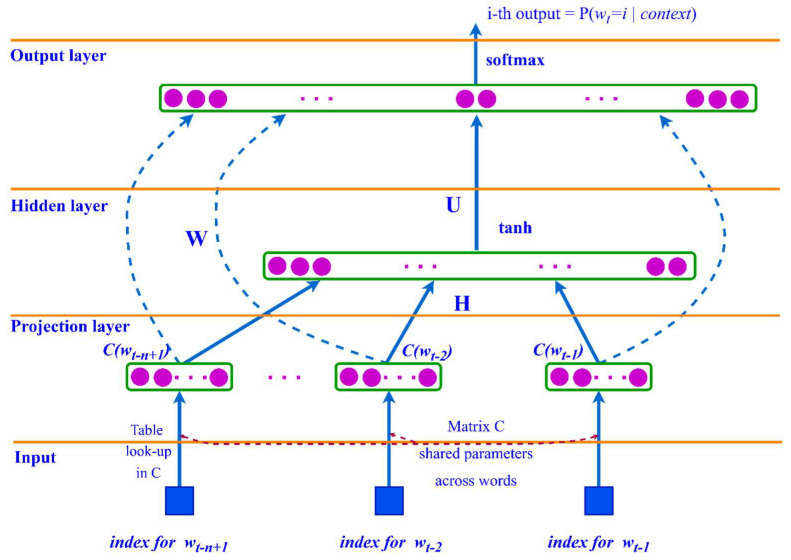
FFNN language model architecture.

The architecture of the FFNN language model is shown in Figure 4 and can be described as follows:

(9) $$\begin{array}{c} y=b+Wx+Utanh\left(d+Hx\right), \end{array}$$

where *H, U*, and *W* are the weight matrices for the connections between layers, and *d* and *b* are the bias values of the hidden and output layers, respectively.

## 4. ANN Architecture for Language Modeling

However, this model has several limitations. Before training the network, the specified context was limited in terms of length. The FFNN does not use time information to model the language model. In addition, a fully connected neural network must learn several learning parameters. Although the number of such parameters is lower than that of an N-gram, such a network is still considered ineffective.

**Figure 5:**
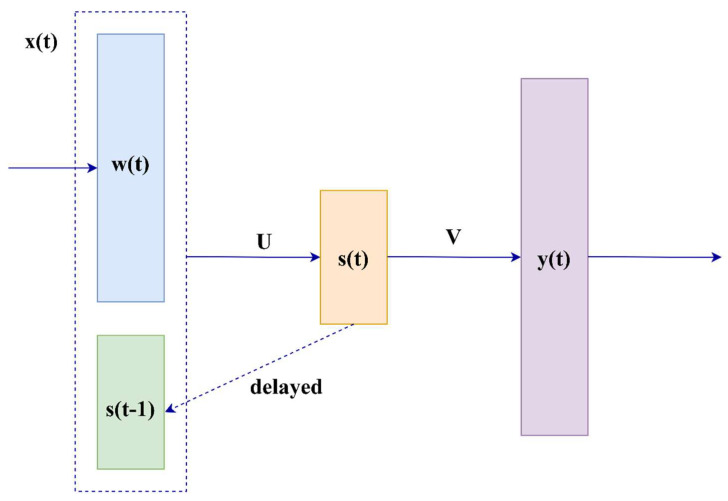
Architecture of language model based on recurrent neural networks.

RNN language models. The idea of using RNNs to create a language model was first proposed in [50]. The architecture of the originally proposed RNN language model is illustrated in Figure 5, and recurrent neural network language modeling (RNNLM) at t-step can be described as follows:

An RNN has an internal state that changes at each step depending on the previous context. The state vector *S_t_* can be obtained from the word *w_t_* and state *s_t−1_*,

(10) $$\begin{array}{c} x_{t}={\left[\omega_{t}^{T};s_{t-1}^{T}\right]}^{T}, \end{array}$$

(11) $$\begin{array}{c} s_{t}=f\left(Ux_{t}+b\right), \end{array}$$

(12) $$\begin{array}{c} y_{t}=g\left(Vs_{t}+d\right), \end{array}$$

where *U*, *W*, and *V* are weight matrices; *b* and *d* are the state layer outputs and output layer bias values, respectively; *f* is a sigmoid function; and *g* is a softmax function.

Although RNN language models can use all contexts for prediction, it is difficult to train them to learn long-term relationships because the parameter gradients may disappear or increase during RNN training. This leads to a slow reading or infinite parameter values [51]. The use of recurrent networks with LSTM has solved this problem. 

LSTM-RNN language models. The use of LSTM in language modeling was first considered in [49]. With the exception of the memory block and a part of the neural network, the architecture of LSTM-RNNLM is almost the same as that of RNN-LM [52].

Although the LSTM-RNN language model performs well, it takes a long time to train the model on a large corpus because the distribution of the predicted words is explicitly normalized through the softmax layer. This causes all letters in a word to be considered when calculating the log-probability gradients. Researchers are still exploring various ways to improve the language models applied in neural networks. Within the framework of this study [53], various ways to organize a language model at the level of the letters (character-level models) have been considered. The authors of [54] considered factorial language models, that is, ways to form a language model based on the features of the word form (beginning with uppercase or lowercase letters, affixes, hyphens, and other factors). In [55,56], the problem of creating two-sided (whether the chosen word depends not only on those words that come before it, but also on those that come after it, is considered) language models for calculating the probability of a word was considered. Methods for developing attention-based language models were also presented in [57,58].

**Creation of a language model and its integration into a speech recognition system.**

Each sentence in the body text is enclosed between the characters <s> and </s>. Thus, when building a language model, the beginning and end of the text are clearly indicated. The text corpus prepared to create a language model is as follows.

*<s> bugun havo harorati yuqori bo’ladi </s>*

*<s> bugun havo issiq bo’ladi </s>*

*<s> ... ... ...</s>*

Each sentence (text) is written on a separate line. After the text corpus is formed, we can create a language model.

**Creation of a statistical language model**. We developed a three-gram language model using the Kneser–Ney smoothing method for an N-gram language model.

Special tools are available for creating a language model of N-grams. Examples include KenLM [59] and IRSTLM [60] toolkits. This study uses the IRSTLM tool.

The ARPA N-gram format is the standard used by many decoders for the N-gram model. All probabilities and weights are presented in log10. The first part of the file is a header comment and is ignored by the decoder. In an ARPA file, the language model information is presented as follows.

*\data\*

*ngram 1 = 5*

*ngram 2 = 12*

*ngram 3 = 21*

*\1-grams:*

*log10_prob(word1) word1 log10_backoff(word1)*

*log10_prob(word2) word2 log10_backoff(word2)*

 *...*

*\2-grams:*

*log10_prob(word1|word1) word1 log10_backoff(word1,word1)*

*log10_prob(word1|word2) word2 word1 log10_backoff(word2,word1)*

*...*

*\3-grams:*

*log10_prob(word3|word1,word2) word1 word2 word3*

*...*

*\end\*

The main purpose of using language models in the field of speech recognition is to determine the probability of finding text, as proposed by a speech recognition system. For instance, let us consider calculating the probability of the sentence “*bugun havo issiq*” using a language model.

*P(“bugun havo issiq”) = P(“bugun havo issiq”) + P(“havo issiq”) + P(“issiq”)*

**Figure 6:**
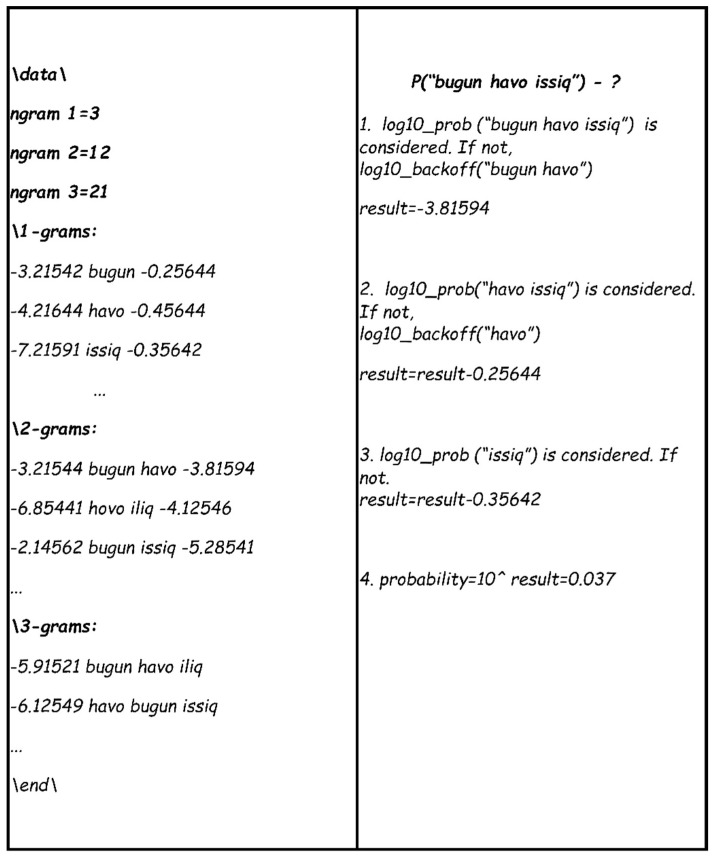
Computing the probability of a sentence using the language model of N-grams.

The probability of a given offer is calculated through the following expression: However, such sequences may not exist in the language model, which can be solved as follows in Figure 6.

**Language model based on neural networks.** Based on this approach, we will be able to correct for the main drawback of the N-gram language model. As the main disadvantage of the N-gram model, it divides a long sequence of contexts into small N-grams. In such cases, the possibility of relatedness of words located far from each other is not considered. In addition, as N increases in the N-gram model, the size of the model also increases. Thus, the use of neural language models is effective at filling these gaps. The RNN recurrent network achieves satisfactory results in identifying short and long links in a text.

**Figure 7:**
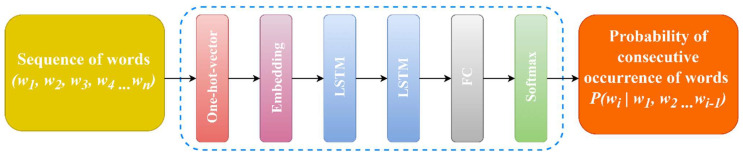
Architecture of neural network language model.

Based on the size of the created text base, the following network architecture is proposed: The structure of the LSTM-LM network is illustrated in Figure 7.

**Network architecture.** The input to the network is the sequence of words in each sentence in the text corpus. Initially, the words are numbered according to the dictionary. They are then encoded using a hot vector method based on the dictionary size. Consider a one-hot method, with a dictionary size of V = 3 words, code error word *“bugun”* word index = 2, single vector [0,1,0], word index = 3, and single vector [0,0,1].

In the next step, the embedding layer has a size of [V,300]. A 20% dropout layer is used after the embedding layer. A 15% dropout layer is used after two consecutive LSTM layers with 650 memory cells, with a 25% dropout between them. Finally, with a softmax activation layer, |V| has a fully bounded layer size. The network parameters of the proposed LSTM-based neural language model architecture are presented in Table 1.

## 5. Experiments on Uzbek Continuous Speech Recognition Based on the Proposed Neural Networks Using LM

### 5.1. RNN-Based LM Architecture for Continuous Uzbek Speech Recognition 

**Figure 8:**
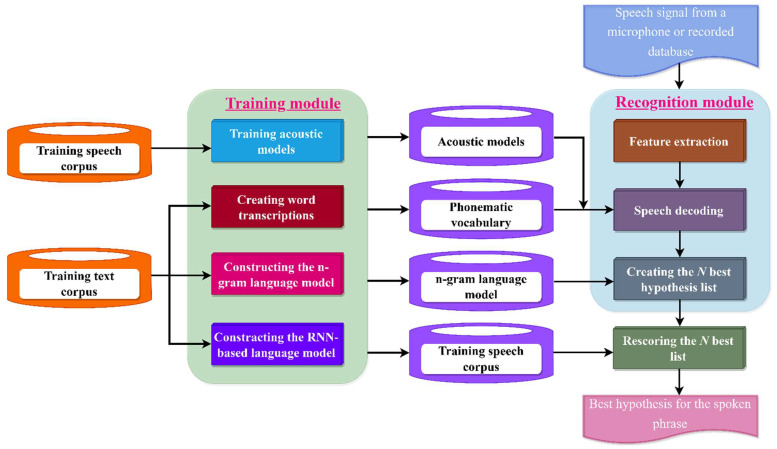
Architecture of RNN- and LM-based recognition system for continuous Uzbek speech.

The construction of the RNN-based LMs-based automatic continuous Uzbek speech-recognition system is shown in Figure 8. The system supports both training and recognition modes of operation. Models for acoustic speech units, a phonetic lexicon of word forms, N-grams, and a neural network LM were built in training mode. The input speech signal is transformed into a sequence of feature vectors in recognition mode (using Mel-frequency cepstral coefficients and their two derivatives). The previously taught auditory and linguistic models were then used to assess the most plausible hypothesis. Rescoring the N-best lists is done post-processing using the RNN-based LM. The best idea for the recognized sentence is then selected by rescoring the generated ideas using the RNN-based LM.

### 5.2. Training and Test Speech Corpus 

Approximately 80 million words from various sources were used to build the Uzbek language model. The number of unique words in this corpus was approximately 68 thousand, and 15 million sentences (sentences) were collected during the corpus construction. All collected sentences were transferred to the Uzbek Latin alphabet in Table 2. The initial normalization steps were applied. Experiments were conducted to create a language model of the Uzbek language based on N-grams and neural networks. We used the open-source SRILM toolkit to build an N-gram language model as well as the TensorFlow and Keras packages to build our neural network-based language models. The capabilities of the two language models were evaluated both separately and jointly.

### 5.3. Experimental Results of Using RNN-Based LM in a Continuous Uzbek Speech Recognition System

Despite being based on statistics, the N-gram language model, as discussed above, is used in many applications today. Therefore, we conducted several studies on N-gram language models.

The N-gram language model improves the performance by applying soothing and back-off operations directly. 

Flexibility and perplexity were used to evaluate the created language model. This is the inverse probability of the test set, normalized by the number of words in the set, and is calculated as follows:

(13) $$\begin{array}{c} PP\left(W\right)=P{\left(w_{1}w_{2}\ldots w_{N}\right)}^{-\frac{1}{N}}=\sqrt[{N}]{\prod_{i=1}^{N}\frac{1}{P\left(w_{i}|w_{i}\ldots w_{i-1}\right)}}, \end{array}$$

where *W* is the sequence of words, and *N* is the number of words in the test set.

This chain rule can be used to increase the probability of *W*. Then, (10) has the following form:

(14) $$\begin{array}{c} PP\left(W\right)=\sqrt[{N}]{\prod_{i=1}^{N}\frac{1}{P\left(w_{i}|w_{i}\ldots w_{i-1}\right)}}. \end{array}$$

It can be seen from the above expressions that the lower the bewilderment that occurs, the higher the efficiency of the language model.

**Figure 9:**
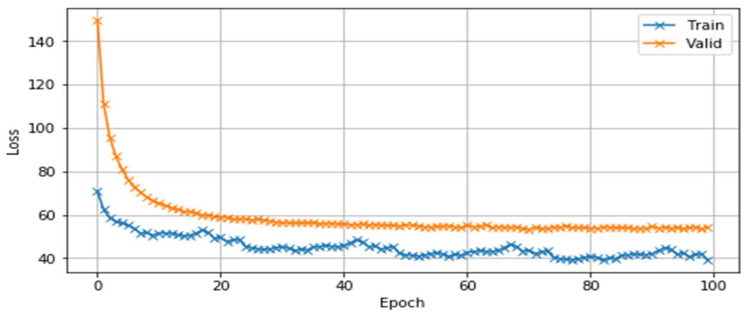
Change in loss error during learning steps sizes.

**Figure 10:**
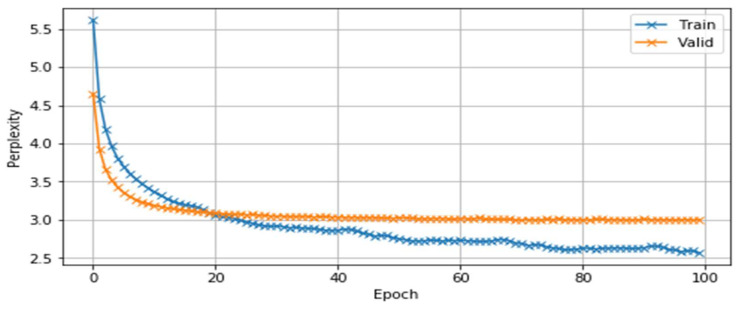
Changing perplexity of the model during learning stages.

It can be seen that when the three-gram language model was built using the IRSTLM statistical language model tool, the *perplexity* values for the *training* and *test* set were 7.2 and 8.9, respectively. In the language model based on a neural network, the values were 2.56 for the training set and 3.12 for the test set. Figure 9 and Figure 10 show the changes in loss and *perplexity* values during the training iterations of the neural-network-based language models.

We separated 4000 sentences from the collected text corpus into a test set to evaluate the language model. Using Kneser–Ney and Katz types of smoothing, we built 3-,5-,7-, and 9-grams and calculated the perplexity value, as shown in Table 3.

We can see in the table that because we use the back-off algorithm, the perplexity indicator improves with an increase in N-gram orders. In addition, with the ability to interpolate, Kneser–Ney performs better than Katz smoothing. Word- and character-based types of neural network language models have been developed. First, for comparison with N-gram language models, we compared simple Vanilla RNN and LSTM language models when considering word-based experiments. The length of the output layer for these two networks was equal to the number of unique words in the L-corpus, as shown in Table 4.

The LSTM language model shows a significantly different performance compared with the N-gram language model. If we use the LSTM network, the problem of the limit of the sequence of words in the N-gram can be solved. The word-based LSTM language model achieved a lower classification performance because the length of the output layer was excessively large. However, in a character-based language model, the length of the output layer is small. Therefore, the classification ability is good. There are 27 characters in the Uzbek Latin alphabet (several letters, such as s + h = sh, n + g = ng, are not counted). If we also take into account spaces (“_”), characters representing the beginning and end of a line (<s>,</s>), and characters representing an unknown character (<unk>), there will be a total of 31 output neurons. We evaluated the effectiveness of building word- and character-based language models from the LSTM neural network, the results of which are presented in Table 5.

In conclusion, it should be stated that character-based language models are more effective than word-based language models.

Within the framework of the study, the process of integrating the developed language model into an automatic recognition system of Uzbek speech and analyzing the accuracy of the recognition was developed. To achieve a speech recognition system for acoustic modeling, a 105 h speech corpus and several integrated neural network models (E2E-LSTM, RNN-CTC, DNN-HMM, E2E-Transformer, and E2E-Conformer) have been used. For instance, as a result of learning the E2E-Conformer model, the speech recognition system achieved a WER of 18.1% and a CER of 7.8%, whereas for the test set, a WER of 17.4% and a CER of 5.8% were achieved. 

By combining the developed language model with the speech recognition system, we achieve a WER of 15.1% and a CER of 5.5% for the train sample, and a WER of 13.9% and a CER of 5.26% for the test sample.

The results obtained by combining the language model with the other models are presented in Table 6.

When the created language model is applied to the speech recognition system, we can see the results in the table below (examples from the conducted experiments are given). In this case, the first column (Original text) contains the audio text read to the speech recognition system, and the second column (Recognized text) includes the recognized text based on the application of the language model to the speech recognition system. The rest of the columns are evaluated according to the speech recognition system’s word error rate (WER) and character error rate (CER) metrics (Table 7).

## 6. Conclusions and Future Work

Generating a large corpus of text is one of the most important and complex tasks in the creation of a language model. It is impossible to create suitable search robots or scripts for the stages of a transformation, or to clean the data and resolve all data collection tasks from open Internet systems. It is therefore necessary to determine the individual solutions for each stage.

As part of this research, language models were created based on statistics and neural networks to recognize continuous speech in the Uzbek language. The perplexity indicator in the generated language models was 7.2 in the three-gram language model and 2.5 in the LSTM-LM language model. As a result of an integration of the created language models into an automatic speech recognition system, it was proven that the recognition accuracy increased significantly. The developed language model can be used in applications designed to solve other problems in computational linguistics, in addition to speech recognition systems.

In this reviewed research work, research was carried out on the creation of language models based on statistics and neural networks and their effectiveness in speech recognition. The volume of Uzbek textual data (dataset) in building language models is not very large. Additionally, the use of the language models created in the work is considered only for improving the efficiency of speech recognition systems, and in fact, language models can be used to solve other types of problems. These include natural language processing, natural language understanding, natural language generation, machine translation, etc. In our future work, we will conduct research on the application of the language model in the above-mentioned areas. For this, we will first need to collect a large amount of Uzbek language data. It is necessary to create crawler programs that facilitate the automatic collection of textual information from various sources. Additionally, it is our future goal to master other types of technologies designed to build language models and use them to build a more effective language model. 

## Figures and Tables

**Table 1:** Network parameters.

№	Name of Layer	Parameters of Layer	Number of Layers
**1**	Input layer	One-hot vector based word sequence	1
**2**	Embedding layer	Size of layer = [|V|,300] Activation function = RELU, Dropout = 20%	1
**3**	Recurrent layer	Type of RNN = MultiCell LSTM, Number of memory cells = 650, Activation function = RELU, Dropout = 20%	2
**4**	Fully bonded layer	Size of layer = |V|, Activation function = softmax	1
**5**	Learning Options	Optimization algorithm: Adam, Learning stride length = 0.001, size of Batch = 30	

**Table 2:** Specifications of the Uzbek language corpus dataset.

CORPUS	Total Number of Words	Total Number of Unique Words	Total Number of Sentences
**Test Data**	16 M	13.6 K	3 M
**Train Data**	64 M	54.4 K	12 M
**Total**	80 M	68 K	15 M

**Table 3:** Calculation of the perplexity value of 3-, 5-, 7-, and 9-grams using the Kneser–Ney- and Katz-type smoothing.

N-Gram Model Baseline, Soothing Type	N-Gram Order	Perplexity	
		Training Set	Test Set
**Kneser–Ney + back-off**	3	142.1	121.2
	5	134.3	114.5
	7	128.8	112.3
	9	122.2	108.1
**Katz + back-off**	3	165.5	132.3
	5	151.2	123.8
	7	145.9	119.3
	9	139.4	112.1

**Table 4:** Perplexity of vanilla RNN and LSTM language models.

Type of RNN	Perplexity	
	Training Set	Test Set
**Vanilla RNN**	76.4	63.6
**LSTM**	62.3	51.4

**Table 5:** Evaluating the effectiveness of building a word- and character-based language model from LSTM neural network.

Type of LM	Perplexity	
	Training Set	Testing Set
**Word-Based**	62.1	51.2
**Character-Based**	7.2	4.7

**Table 6:** Accuracy rates based on models.

Model	Character Based LM	SP	SA	Valid		Test	
				CER	WER	CER	WER
**E2E-LSTM**	✗	✗	✗	13.8	43.1	14.0	44.0
	✗	✗	✗	14.9	30.0	14.3	31.4
	✗	✓	✗	13.7	27.6	14.4	30.6
	✗	✓	✓	12.6	24.9	12.0	27.0
	✓	✓	✓	10.5	21.7	11.1	23.2
**DNN-HMM**	✗	✗	✗	12.8	34.7	10.2	32.1
	✗	✗	✗	10.3	20.5	8.6	24.9
	✗	✓	✗	6.9	18.8	7.5	23.5
	✗	✓	✓	6.9	19.9	8.1	24.9
	✓	✓	✓	5.2	16.4	6.0	21.3
**RNN-CTC**	✗	✗	✗	13.3	35.8	9.7	32.3
	✗	✗	✗	12.2	27.2	9.1	24.3
	✗	✓	✗	10.9	25.1	8.7	23.9
	✗	✓	✓	8.3	24.7	7.9	22.3
	✓	✓	✓	5.9	22.7	6.9	20.8
**E2E-Transformer**	✗	✗	✗	12.3	35.2	9.4	31.6
	✗	✗	✗	11.7	25.7	8.7	23.9
	✗	✓	✗	10.7	23.9	8.4	23.0
	✗	✓	✓	9.9	21.4	7.6	21.0
	✓	✓	✓	5.9	19.3	6.0	18.9
**E2E-Conformer**	✗	✗	✗	12.7	37.6	10.7	35.1
	✗	✗	✗	11.5	27.5	9.7	26.3
	✗	✓	✗	9.2	21.7	7.5	21.2
	✗	✓	✓	7.8	18.1	5.8	17.4
	✓	✓	✓	**5.5**	**15.1**	**5.26**	**13.9**

**Table 7:** Examples of the application of the created language model to the speech recognition system.

N	Original Text	Recognized Text	WER	CER
**1**	Davlat qonunchiligiga ko’ra barcha bepul ta’lim olish xuquqiga ega	Davlat qonunchiligiga ko’ra bacha bepul ta’lim olish xuquq ega	11%	1.5%
**2**	ilova hozircha faqat ios dasturlarida ishlaydi android versiyasi ishlab chiqish jarayonida	ilm va hozircha faqat ios dasturlarida ishlaydi andro versiyasi ishlab chiqish jarayonida	27.2%	4.5%
**3**	qonun oldida barcha teng	qon oldinda barcha teng	50%	13%
**4**	yakka tartibdagi tadbirkor davlat ro’yxatidan o’tkazilganligi to’g’risida guvohnoma beriladi	yakka tartibdagi tadbirkor davlat ro’yxatidan o’tkazilganligi to’g’risida guvohnoma beriladi	0	0
